# Training frontline workforce on psychosis management: a prospective study of training effects

**DOI:** 10.1186/s13033-015-0029-3

**Published:** 2015-11-19

**Authors:** Tore Sørlie, Marit Borg, Karin B. Flage, Ole-Bjørn Kolbjørnsrud, Gunnar B. Haugen, Jūratė Šaltytė Benth, Torleif Ruud

**Affiliations:** Department of Clinical Psychiatry, University of Tromsø-The Arctic University of Norway, Tromsø, Norway; Department of Mental Health and Substance Abuse, University Hospital of North Norway, Tromsø, Norway; Buskerud and Vestfold University College, Drammen, Norway; Centre for Psychotherapy and Psychosocial Rehabilitation of Psychoses (SEPREP Foundation), Oslo, Norway; Telemark Hospital, Notodden/Seljord District Psychiatric Center, Notodden, Norway; HØKH, Research Centre, Akershus University Hospital, Lørenskog, Norway; Institute of Clinical Medicine, University of Oslo, Oslo, Norway; Division of Mental Health Services, Akershus University Hospital, Lørenskog, Norway

**Keywords:** Evaluation, Multidisciplinary cooperation, Psychosis management, Staff training

## Abstract

**Background:**

The care situation for persons experiencing severe mental illness is often complex and demands good coordination, communication, and interpersonal relationships among those involved from the primary and specialized mental health care systems. For 15 years, professional care providers from different service levels within the same geographical areas in Norway have been trained together in a 2-year local onsite training program with the aim of increasing skills, joint understanding, and collaboration in their work with individuals experiencing severe mental illness.

**Methods:**

The key aspects of competence addressed by the training program were measured at baseline, after 1 year, and at the end of the training period. Professional education and experience were also rated at baseline. Data were collected between 1999 and 2005 and were analyzed by estimating a linear mixed model.

**Results:**

Results showed a significant increase in participants’ experienced competence in all training goals, especially for the understanding of psychosis and relationship building. There was no significant variance at the program level, indicating consistent implementation of local programs.

**Conclusions:**

This prospective study indicates that the training program was successful in increasing perceived competence in the areas addressed, and training staff from different service levels together probably contributed to more collaboration. This training model still operates in Norway.

## Background

In the current era of deinstitutionalization, people experiencing long-term severe mental illness are increasingly relegated to living at home where they often have to cope with various socioeconomic challenges. Complex care situations demand good coordination, communication, and interpersonal relationships among providers from primary and specialized mental care systems [1] and represent one of the greatest challenges to solving the dilemma of obtaining a sufficient mental health workforce [2]. The World Health Organization is urging countries to direct resources toward the development of robust primary health care services to improve access to mental health treatment [3]. There has also been an emphasis on the importance of functional treatment networks that include both primary and specialized care. This implies a need for collaborative training programs for mental health professionals who provide interdisciplinary services across agencies that offer adequate treatment, rehabilitation, and support for citizens living with severe mental health challenges [1, 4]. One of the ideas underlying the training program described here is that collaboration among participants may be developed, learned, and improved through common training contexts. Another central aspect of the learning philosophy is that service users and family members are involved in program development and in training sessions. This ensures that learning is more easily transferred to real life situations. Thus, this training model focuses on the needs of service users, health care providers, and the health services.

Studies of the salient components of effective mental health skills training programs highlight the importance of the following factors: the use of small groups of trainees led by expert facilitators or supervisors who meet regularly over extended periods of time to discuss cases, the use of interactive learning, and the inclusion of performance feedback [5]. Other emphasizes the importance of a facilitative learning environment [6] and the sharing of emotions arising from clinical experiences [7]. The latter may have relevance for both the learning process and the wellbeing of professionals [8]. Training programs that systematically focus on students’ own working experiences may increase motivation because they feature relevant personal experiences and provide an opportunity for students to master their needs. The training program described here included long-term small group clinical supervision with an emphasis on experiential material.

### The training program

The Center for Psychotherapy and Psychosocial Rehabilitation of Psychoses (SEPREP) was founded in 1990 to improve treatment and care for people who are experiencing severe mental health problems. SEPREP’s main activity is training professionals.

The goals of the training program, which still operates, are as follows: (1) better understanding of psychosis, psychosocial rehabilitation, and treatment; (2) improved skills in building and maintaining relationships with persons in need of services; (3) improved awareness of interactions with those persons to better understand their experiences and needs; (4) improved participation in multidisciplinary collaboration; (5) improved teamwork and collaboration across service levels; (6) increased support of carers; and (7) improved ability to take care of oneself while performing clinical work.

This 2-year training program consists of lectures 1 day a month (80 h), supervision in small multidisciplinary groups every 2 weeks (80 h), discussion and reflective work on theory in small groups once a month (40 h), and writing two clinical case stories integrating theory and literature (group task). The lecturers are clinicians, researchers, and users are involved in about 20 % of the lectures. The supervisors are clinicians with extensive experience in the treatment of people with severe mental illness and in the provision of clinical supervision. Participation in the training is part-time while the students continue in their clinical job, facilitating interactions between training and practice. Each supervision group consists of health workers from both community mental health centers (CMHCs) and primary care providers. By training the workforce together, the program offers a combination of individual training and system-level intervention. The programs are planned and implemented locally in cooperation between representatives of a CMHC, collaborating municipalities, users and SEPREP.

This article will report on the effects of the training program in accordance with its learning goals.

## Methods

### Research questions

1. Is there an increase in experienced individual competence in relation to the training goals of the program?
2. Are patterns of change in competence different across training goals? How do service level and previous clinical experience influence the patterns of change in experienced individual competence during the training program?
3. Are changes in experienced individual competence different across local training programs, and if so, what factors may explain the differences?

### Design and procedure

Prospective longitudinal questionnaire based cohort study. Changes in participants’ experienced competences related to the individual program goals were assessed at the start of the program, after 1 year, and at the end of the 2-year program. Each participant was asked to include on their anonymous questionnaire a personal id-number consisting of the last two digits of their mother’s birth year and the first two letters of their mother’s first name. This enabled questionnaires from the same persons to be kept anonymous and still be linked across the three data collection times, as this information was expected to be reliably recreated each time. Data collection took place between 1999 and 2005. Since the evaluation form was changed in 2005, later evaluation data could not be included in the present study.

### Ethics

The study was not based on individual sensitive material. Thus, approval from the Regional Ethics Committee was not necessary.

### Materials

Data consisted of 1846 questionnaires from 56 local programs at the start of the training, 1492 from 55 programs after 1 year, and 1258 from 53 programs at the end of the 2-year training. Based on the data from 55 local programs, the average response rate compared with the number who started the training was 93 % at the start of the program, 83 % after 1 year, and 72 % after 2 years. The completion of questionnaires during program sessions contributed to the high response rates. It is likely that the response rate decreased over time mainly because some participants left the programs.

46 % of the participants were from primary care and 54 % from CMHCs. 20 % had university educations (6 years), 67 % had college educations (3 years), and 4 % had 1-year professional educations. 20 % had little clinical experience with severely mentally ill people, 33 % had some, 25 % had substantial experience, and 14 % had a moderate amount of clinical experience. 10 % of the sample was aged 20–29 years old, 35 % was aged 30–39, 22 % was aged 40–49, and 33 % was 50 or older.

### Questionnaire and variables

The questionnaire was designed to measure experienced competence in relation to the seven goals of the training program. Each of the seven subscales related to these goals contained three to 10 items, each describing an aspect of the specific skill to be rated on a five-point scale. Principal component analysis of a large sample of completed questionnaires at an early phase of the program (842 at the start, 470 after 1 year, and 207 at the end of the 2-year training) yielded factors in agreement with the competences and indexes based on these factors that showed acceptable internal consistency. The inter-item reliability (Cronbach’s alpha) was acceptable for all subscales. Face validity of the subscales was also acceptable, with most items being correctly assigned to their respective subscales by a group of 39 health workers or researchers who were unfamiliar with the questionnaire and not taking part in the program. Factors, items, inter-item reliability (Cronbach’ alpha) as well as the percentages of correctly assigned items (face validity) are shown in Table 1.

**Table 1:** Content of subscales based on factor analysis of the questionnaire

Subscale (with Cronbach’s alpha/year) and items^a^ in subscale	Factor loadings			Face validity^b^
	Year 0	Year 1	Year 2	
	N = 842	N = 470	N = 207	%
*(1) Understanding psychoses and treatment (alpha: 0.90; 0.80; 0.86)*				
3. Understand much of the experience of having a psychosis	0.76	0.73	0.73	84
4. Know a good deal about how different causes may contribute to psychosis	0.81	0.81	0.79	100
5. Know a good deal about how different severe mental disorders appear	0.80	0.77	0.78	100
6. Understand psychosis from a psychological development perspective	0.78	0.73	0.77	100
7. Know how medical and psychosocial treatments should be used for psychosis	0.65	0.71	0.72	95
8. Know what tasks I have in the treatment of patients with severe mental illness	0.66	0.50	0.52	39
9. Have adequate knowledge and training for treatments I may contribute to	0.64	0.46	0.52	63
10. Have a clear picture of psychotherapy for patients with a psychosis	0.74	0.54	0.55	89
*(2) Building and maintaining relationships with patients (alpha: 0.83; 0.78; 0.75)*				
16. Uncertain how to establish a confident relationship with a psychotic patient	0.59	0.66	0.62	89
17. Feel confident in how to keep and develop my contact with a psychotic patient	0.71	0.75	0.58	95
18. Fully understand how to relate to a psychotic patient	0.76	0.73	0.55	87
*(3) Using own feelings and reactions to understand patients (alpha: 0.66; 0.49; 0.67)*				
12. Often aware of my own feelings while observing and interacting with patients	0.79	0.68	0.75	92
13. My reactions to patients often help me to understand how to relate to them	0.75	0.68	0.79	92
15. Emotional reactions of different staff can help my understanding of the patient	0.67	0.66	0.71	55
*(4) Participating in multidisciplinary collaboration (alpha: 0.66; 0.66; 0.65)*				
21. Experience myself as able to contribute greatly in multidisciplinary collaboration	0.62	0.45	0.53	87
22. Have many thoughts that I do not express in multidisciplinary collaboration	0.71	0.75	0.62	61
23. Unclear picture of my role or tasks in multidisciplinary collaboration	0.75	0.77	0.73	87
24. Have good experiences of multidisciplinary collaboration for persons with psychosis	0.53	0.36	0.58	89
*(5) Teamwork and collaboration (alpha: 0.64; 0.45; 0.65)*				
25. Fully aware of who my local coworkers are in relation to patients with psychosis	0.48	0.12	0.47	76
26. In this area, we usually do not collaborate across services regarding psychoses	0.59	0.01	0.68	74
27. Psychotic patients have several needs for which there is no local competence	0.63	0.73	0.55	29
28. Easy to ask other collaborators for advice regarding patients with psychosis	0.60	0.39	0.64	89
29. Many collaborators do not know how my team works with psychotic patients	0.65	0.69	0.66	87
*(6) Supporting family/relatives of patients (alpha: 0.58; 0.77; 0.76)*				
30. Where I work, it is not typical to collaborate with relatives of psychotic patients	0.59	0.79	0.77	89
32. Where I work, relatives of patients in the acute phase get little information	0.79	0.79	0.75	97
33. Where I work, we try to give relatives of psychotic patients support and care	0.76	0.82	0.79	100
34. We usually inform relatives about local organizations for relatives	0.42	0.60	0.66	100
*(7) Taking care of myself (alpha: 0.57; 0.63; 0.59)*				
35. Feel content in my work	0.52	0.56	0.60	95
37. Do not have opportunities to share my feelings regarding patients with my colleagues	0.59	0.62	0.59	63
38. Make sure to take breaks when I am tired	0.69	0.72	0.71	100
39. Make sure to get supervision regarding patients I have problems relating to	0.73	0.73	0.56	63

^a^Scale for items was from 1 = not true to 5 = true. Ratings of negatively phrased items were converted for analysis

^b^Assignment of items to subscales for testing face validity was carried out by 38 health workers or researchers unfamiliar with the questionnaire. For this test, the sequence of the items was rearranged so that items from each subscale were spread throughout the questionnaire

### Statistical analyses

Multilevel data analysis was performed by estimating a linear mixed effects model [9] for each subscale (SAS PROC MIXED procedure). The normality assumption was assessed by examining the histograms. First, an unconditional growth model was estimated to assess a time trend in the dependent variable. A second-order time component was included in the model where significant. Next, bivariate analyses with person- and program-level covariates as fixed effects were performed. The interactions between person-level covariates (sex, age, working place, education, clinical experience with the severely mentally ill) and time were tested and left in the model if significant. Finally, the multivariate linear mixed model with time components and person- and program-level covariates (size of program, proportion from primary care, dropout rate) was estimated. The models containing both fixed and random effects were estimated using unstructured covariance. If significant, random effects for both intercepts and time on person- and program-level were included. Subscale 7 (taking care of oneself) was excluded from the multilevel analysis because it was not considered a treatment competence and because there were no significant differences across the three points in time. To avoid hypothesis fishing, data splitting was employed [10]. The data were randomly split into two parts by local programs, with all data from one local program used in the same part. The first data set with 30 % of cases was used to identify a model. The second data set was used to test the model. Only those relationships that were significant in both parts of the data were considered significant when fitting the model to the entire data set. The results are presented as regression coefficients estimated on the entire data set, each with specified level of significance. Model assumptions were tested by standard methods. Effect sizes are reported as regression coefficients (R^2^). The analyses were performed using SAS version 9.2 (SAS Institute Inc., Cary, NC, USA).

## Results

Results are provided in Table 2. There was a significant increase in experienced individual competence for all training goals. The increase was quite large and significant (p < 0.01) for the understanding of lived experiences of psychosis and relationship building (subscales 1 and 2, respectively), with smaller but still significant (p < 0.01) increases for awareness of own reactions (subscale 3), multidisciplinary work (subscale 4), teamwork (subscale 5), and family and carer support (subscale 6). There was a significant second-order time component for the two first subscales (understanding psychoses and relationship building). This indicates a rapid increase in experienced competence during the first year of training, with a weaker change during the second year. The increase in the first two subscales was greatest among those with less experienced competence at baseline (both p < 0.01). The same pattern was found in the first subscale for participants from primary care as compared with those from CMHCs (p < 0.05). The multilevel regression analysis did not show any significant differences in experienced competence across the local programs, and the program-level variables (size of program, proportion from primary care, dropout rate).

**Table 2:** Results of linear mixed models for changes in experienced competence and predictors (regression coefficients: R^2^)

Subscales	Subscale 1	Subscale 2	Subscale 3	Subscale 4	Subscale 5	Subscale 6
	Understanding psychoses	Building relationships	Using own reactions	Multidisciplinary collaboration	Teamwork and collaboration	Supporting relatives
Intercept	2.5840**	2.7907**	3.4252**	3.7106**	3.3962**	2.9464**
Years in training	0.7636**	0.6541**	0.1681**	0.1981**	0.1219**	0.1151**
Years in training × years in training	−0.1311**	−0.0870**				
Individual variables						
Age group	0.0130 ns	−0.0179 ns	−0.0076 ns	−0.0083 ns	0.0285 ns	0.0369 ns
Service level (primary/specialized)	0.1897**	0.0749**	0.0403 ns	0.0142 ns	0.0384 ns	0.1609**
Profession	−0.0465*	−0.0242 ns	−0.0610 ns	−0.0798**	0.0162 ns	−0.0536 ns
Length of professional education	−0.3412**	−0.2107**	−0.1328**	−0.1029*	−0.0882*	−0.1421**
Clinical experience with SMI	0.2851**	0.3270**	0.0640 ns	0.0947**	0.0468**	0.0603 ns
Years in training × service level	−0.0792*					
Years in training × education	0.1040**					
Years in training × clinical experience	−0.0752**	−0.0966**				
Local program variables						
Number of students	−0.0041 ns	0.0057 ns	0.0017 ns	0.0022 ns	0.0041 ns	0.0122 ns
Portion from primary care	−0.0022 ns	−0.0033 ns	−0.0012 ns	−0.0009 ns	−0.0004 ns	−0.0017 ns
Dropout rate	0.0022 ns	0.0019 ns	0.0013 ns	−0.0029 ns	−0.0017 ns	0.0008 ns
Proportion of total variance explained by individual factors						
Intercept (%)	58.4	43.4	9.7	9.0	31.5	26.8
Slopes (%)	45.3	21.7	0.7	−10.0	No random slopes	No random slopes

Levels of significance: * p < 0.05, ** p < 0.01

R^2^ = regression coefficients = explained variance

*ns* not significant

## Discussion

A significant increase in experienced individual competence was observed for all training goals; this increase was greatest for understanding of psychosis and relationship building and smaller for awareness of own reactions, multidisciplinary work, teamwork, and family support. As expected, the change was greatest in those with less experienced competence at baseline, particularly among those from primary care, leading to less variation in experienced competence after the training.

One of the ideas underlying this training program is that collaboration among participants may be developed, learned, and improved through common training contexts. The greater improvement in experienced competence for the two first subscales (understanding psychoses and relationship building) during the first year of training among may partly relate to networking effects both within the training program and in the participants’ daily working environment. The greater increase in these two subscales among those with less experienced competence at baseline may also indicate that that it was most new to learn in the first year. We have not been able to find other studies reporting similar findings. Competence in this field is usually considered to be strongly influenced by the quality of professional networks, both in terms of the accessibility of competent coworkers and their support and sharing when caring for individuals experiencing long-term severe mental illness. In line with these considerations, one can also assume that the supervision in small multidisciplinary groups every 2 weeks may have had particular significance for the observed skills development. The significance of clinical supervision on patient and educational outcomes is well documented [11].

The multilevel regression analysis did not show any significant differences in experienced competence across the local programs, and the program-level variables (size of program, proportion from primary care, dropout rate) explained only an insignificant portion of the total variance. The lack of significant variation across local training programs may indicate that they were implemented in a consistent way. The main reasons for this are probably the well-developed manual for organizing and running the local programs and the close collaboration with, and monitoring of, all local programs by regional coordinators from the SEPREP national training program. There is also a joint pool of lecturers and supervisors who are engaged across local programs.

The significant increase in experienced competence revealed in our study is in agreement with external evaluations commissioned by the national health authorities [12, 13]. They found that participants and leaders of services were satisfied with the program and they reported increased clinical competence, improved understanding, and mutual respect between professional groups and employees of different service levels.

This national training program was supported by the Norwegian Action Plan for Mental Health 1999–2008, which shared some of the same goals as a proposed national action plan for workforce development in the United States [14]. The program has a strong emphasis on user and caretaker perspectives, clinical supervision, building of relationships, and improvements in collaboration across services. Reviews of these types of training programs have resulted in several recommended training approaches and best practices [15], including the involvement of users and caretakers in program design [16] and as teachers [17]. In view of these recommendations, the present program could probably put more emphasis on evidence-based practices with regard to user and family member involvement and collaborative work.

This was a prospective study. It was unlikely that the participants were able to remember their initial questionnaire responses across the 1-year interval between measurements. The response rate was high and the results may be considered representative for the whole sample of participants. The last assessment took place at the very end of the 2-year training program. Although participants continued in their usual work during the training, follow-up assessments might have captured participants’ more stable daily work evaluations of their own competencies with no influences from the group processes and mutual sharing experiences in the training program.

This study focused only on changes in participants’ experienced competence. We would have liked to measure how the local training programs influenced clinical practice and collaboration, especially regarding the extent to which patients experienced improvement in services. The issues of clinical practice and collaboration are partly explored in the two mentioned evaluation reports [12, 13]. Resources were not available to measure the patients’ experiences. Another possible limitation of the study is the fact that the data used in the study was collected between 1999 and 2005. However, since the group of participants and the structure, content and pedagogic approach of the program still is very much alike, the representativeness of our results for the current courses is considered large.

Quality of care for the seriously mentally ill is also related to continuity of care. The study did not allow us to evaluate whether participation in the training influenced employment stability. Most likely there is such a correlation. WHO’s policy of improving retention of rural health-care workers recommends that governments ‘design continuing education and professional development programs that meet the needs of rural health workers and that are accessible from where they live and work, so as to support their retention [18].

## Conclusions

The training program has pioneered a model that we have not found elsewhere: training mental health frontline workers from collaborative services together and with systematic involvement of service users and family members to achieve changes on the individual, professional, and systemic level. Our prospective measurement of experienced competence indicates that several of the goals of the training program were reached. Future evaluations should aim to include measurements of fidelity to the training model and possibly measure the impact of training on health workers’ practices, their employment stability, and service users’ experiences.
